# Knowledge and Attitude of the General Population Toward E-cigarette Use in Khyber Pakhtunkhwa: A Cross-Sectional Study

**DOI:** 10.7759/cureus.85704

**Published:** 2025-06-10

**Authors:** Muhammad Haris Khan, Muhammad Ijaz Khan, Samra Israr, Abdul Haseeb, FNU Ariya, Muhammad Ibadullah Khan, Maryam Amir

**Affiliations:** 1 Medicine, Khyber Medical College, Peshawar, PAK; 2 Internal Medicine, Greater Baltimore Medical Center, Towson, USA; 3 Internal Medicine, Medical Teaching Institution-Khyber Teaching Hospital (MTI-KTH), Peshawar, PAK; 4 Medicine, Northwest School of Medicine, Peshawar, PAK

**Keywords:** attitude, e-cigarettes, electronic cigarettes, khyber pakhtunkhwa, knowledge, nicotine, pakistan, practices, public health, vapes

## Abstract

Introduction

Electronic cigarette (e-cigarette) use has risen markedly worldwide in recent years, raising public health concerns. E-cigarettes contain many addictive and dangerous substances, including nicotine, different flavorings, propylene glycol, vegetable glycerin, and others, making them more appealing but, at the same time, have various harmful effects on health. The available literature on e-cigarette and vape use and their adverse effects is insufficient and limited, even in developed countries, let alone in developing nations like Pakistan. This study investigates the knowledge and attitudes of the Khyber Pakhtunkhwa (KPK) population toward e-cigarette use and its adverse effects on human health. This study also disclosed the use of e-cigarettes among different age groups, genders, and socioeconomic classes.

Materials and methods

A cross-sectional survey was carried out from October to December 2023 after obtaining ethical approval from the Institutional Research and Ethical Review Board (IREB). The sample size of our study was 385, which was determined using Cochran's formula. Using non-probability convenience sampling, we collected data from 403 individuals. A pre-validated questionnaire from a study conducted in Karachi was adopted for our study, which had four sections. The first section dealt with demographics, the second with knowledge of using e-cigarettes, and the third and fourth with attitudes and practices around using e-cigarettes, respectively. The chi-square test was used to compare the knowledge, attitudes, and practices (KAP) of e-cigarette users with gender and level of schooling. To assess the socioeconomic status of the participants and ascertain the percentage of e-cigarette users in each socioeconomic class, we also utilized the Kuppuswamy scale, which has been authorized for use in Pakistan.

Results

Of those surveyed, the majority (n=340, 84.4%) knew what e-cigarettes were. Although most respondents (n=228, 56.6%) knew about the various compounds and ingredients in e-cigarettes, they had little idea about the different amounts of nicotine in them. The majority of both men and women were aware of what e-cigarettes were and the various ingredients they included; however, women learned about e-cigarettes from the Internet, and men from friends. Regarding education, a greater percentage of intermediate and bachelor's degree holders knew what an e-cigarette was and what its various parts and ingredients were. The vast majority of participants (n=216, 53.6%) expressed that they "definitely will not" try e-cigarettes, even if a close friend were to ask them to, and they did not believe they would even if their guardian approved. Most participants (n=379, 94%) would not recommend or encourage the usage of e-cigarettes. Only 38 (9.4%) participants in our study acknowledged using e-cigarettes.

Conclusion

This study assessed public knowledge and attitudes toward e-cigarette use. Most individuals lacked adequate knowledge, reflected in their general disapproval. Views were similar across genders, but most users were young adults from higher socioeconomic backgrounds, likely due to greater exposure to marketing and cultural norms. As the first study of its kind in Khyber Pakhtunkhwa, it highlights the need for public education and targeted regulation. Future research should explore the role of e-cigarettes in smoking cessation, usage patterns across demographics, and the impact of marketing on youth.

## Introduction

The use of electronic cigarettes (e-cigarettes) is becoming increasingly prevalent among the general population, particularly among young individuals. E-cigarettes are battery-operated devices that heat and vaporize a liquid, commonly known as e-liquid or vape juice, which may or may not contain nicotine. The resulting aerosol is inhaled by the user, simulating the experience of smoking traditional cigarettes [1]. The World Health Organization refers to electronic cigarettes, also known as e-cigarettes or electronic nicotine delivery systems (ENDS), as having a wide range of addictive and dangerous ingredients, including nicotine, different flavorings, propylene glycol, vegetable glycerin, and others. Nicotine, in particular, is a highly addictive substance, as reflected by the fact that approximately 80% of smokers in the USA who attempt to quit do so unsuccessfully without assistance [2]. In the beginning, e-cigarettes were created and promoted as aids for smokers to stop using traditional tobacco by offering a purportedly safer substitute. However, there is not enough scientific data to definitively indicate that e-cigarettes are successful at helping people quit smoking. Additionally, it has been difficult for authorities to enact and enforce regulations governing the sale, promotion, and usage of these goods due to their novelty and the absence of comprehensive regulatory frameworks. As a result, numerous e-cigarette manufacturers have managed to navigate around existing tobacco control laws [3]. The major selling points for e-cigarette use include its helpfulness in smoking cessation, mitigating the withdrawal effects of nicotine, reducing exposure to the wide variety of carcinogens present in traditional cigarettes, and its affordability compared to combustible tobacco products [4]. E-cigarettes were marketed this way and gained popularity among the general population, particularly convincing the youth to use these products [5].

A survey on middle and high school students in the USA in 2019 revealed that 10.5% of middle school students and about 27.5% of high school students used e-cigarettes [6]. A different UK survey discovered that 14.4% of people used e-cigarettes that contained nicotine [7]. Between 2011 and 2012, studies done in the USA revealed that young adults' use of e-cigarettes increased by 6.8% [8]. A comprehensive analysis found that between 2011 and 2018, the percentage of American youth using e-cigarettes and vaping increased by almost 19% [9]. The major factors contributing to the alarmingly increased use of e-cigarettes are the different types of available e-cigarette devices and, most importantly, the wide variety of flavoring agents, which make them more appealing than traditional combustible cigarettes. According to a US study, e-cigarette flavor was the primary motivator for users to start using them and was also the cause of their addiction [10]. The perception that e-cigarettes are significantly safer than regular cigarettes is another significant contributing factor. According to a US youth research, 73% of teenagers felt that e-cigarettes were less harmful than regular cigarettes, and 47.1% felt that e-cigarettes were less addictive [11].

The rise in popularity of e-cigarettes has been largely attributed to youth- and young-adult-oriented TV commercials and social media efforts. The longitudinal association between exposure to social media e-cigarette advertisements and subsequent e-cigarette use among American adolescents aged 12-17 was investigated in a study conducted in November 2023. According to the study, youngsters are the target audience for e-cigarette marketing methods, especially those that use social media, and exposure to these commercials was linked to higher e-cigarette usage one year later [12]. According to a study conducted in Lebanon, 63.3% of the participants in the survey knew very little about electronic cigarettes, and most of them did not know that they were linked to lung cancer and had negative effects on the heart and brain [13]. According to a study conducted in Karachi, 32% of respondents had a favorable opinion of e-cigarettes and thought they were just as addictive as traditional cigarettes and detrimental to your health [14]. A study conducted in Saudi Arabia investigated the knowledge and attitudes of medical students toward e-cigarettes and found that 31.1% of respondents had a highly favorable attitude, believing that e-cigarette use for smoking cessation could reduce the risk of developing cancer [15]. Another American study looked at medical students' understanding of e-cigarettes. Of the medical students, 35.8% were unaware of whether e-cigarettes were approved by the Food and Drug Administration (FDA) to be used as a helpful aid in quitting smoking, and 4.1% falsely believed that they were [16]. According to 94.8% of the students in the same study, their medical school was not adequately educating them about e-cigarettes [16].

At the time this study was carried out, the US Food and Drug Administration (FDA) did not approve e-cigarettes as a safe substitute for smoking since they have been linked to serious health hazards, including nicotine addiction, respiratory problems, and exposure to dangerous chemicals [17]. Numerous detrimental impacts of vaping and e-cigarettes on human health have been identified by recent studies. It has been suggested that using e-cigarettes impairs cognitive function and development, as research published in 2019 found that e-cigarettes, like traditional cigarettes, can cause oxidative stress, which may negatively impact the brain's normal development; the study also reported that users of e-cigarettes are more likely to experience anxiety, depression, suicidal thoughts, and impaired cognitive function [18]. For some of its users, e-cigarettes may lead to dependence and addiction to other harmful substances, such as cannabis, cocaine, or opioids, which stimulate the same reward system; this progression is referred to as the gateway hypothesis [19]. In 2019, there was a notable rise in e-cigarette or vaping-associated lung injury (EVALI) across all 50 states of the USA. All 50 states in the USA have reported a total of 2,807 cases or deaths to the Centers for Disease Control and Prevention (CDC) as of February 2020 [19]. It is recommended by both the CDC and FDA not to use e-cigarettes or vaping products, given the potential harmful effects these products might have on human health [20].

There is a significant lack of research on vaping and e-cigarette use globally, and this gap is even more pronounced in developing countries such as Pakistan. Within Pakistan, only a handful of studies have examined e-cigarette use, and these are often limited in scope, focusing on specific populations such as healthcare professionals or university students. To date, no research has been conducted on this topic in Khyber Pakhtunkhwa (KPK), making this study the first of its kind in the region. Furthermore, existing studies have not comprehensively examined the epidemiology of e-cigarette use across different age groups, genders, and socioeconomic backgrounds, leaving a critical gap in the national literature. By addressing these gaps, this study not only contributes valuable region-specific data but also provides insights that can guide public health policies and regulatory actions. Ultimately, the findings may help discourage harmful e-cigarette use and support the development of effective legislation to regulate vaping products in Pakistan. Therefore, this study will assess the knowledge, attitudes, and practices (KAP) of e-cigarettes among the general population of Khyber Pakhtunkhwa. Additionally, it will determine the prevalence of e-cigarette use across different age groups, genders, educational levels, and socioeconomic statuses. The chi-square test will be applied to compare knowledge, attitudes, and practices across gender and educational levels to identify any significant associations.

## Materials and methods

A cross-sectional study was carried out from October to December 2023 to investigate the general public's awareness and attitudes toward e-cigarette use in Khyber Pakhtunkhwa. The Institutional Research and Ethical Review Board (IREB) of Khyber Medical College, Peshawar, granted us ethical approval (606/DME/KMC) before we conducted an online survey using Google Forms (Google, Inc., Mountain View, CA), and the questionnaire was disseminated through social media platforms, including Instagram, Facebook, and various WhatsApp groups. We utilized the Strengthening the Reporting of Observational Studies in Epidemiology (STROBE) checklist to ensure the methodological rigor and transparency of our study. This guideline facilitated the accurate reporting of observational research findings. We used non-probability convenience sampling for participant recruitment, and the sample size was determined using Cochran's formula. A sample size of 385 was calculated based on a 95% confidence level, an assumed prevalence of 50%, and a margin of error of ±5%. Informed consent was taken from all the participants, and their anonymity was ensured. A total of 403 individuals filled out our questionnaire completely. Participants from various age groups, genders, and educational levels residing in Khyber Pakhtunkhwa were invited. Our survey had minimal limitations in terms of participant eligibility. The sole inclusion criterion was residency within the province of Khyber Pakhtunkhwa (KPK); individuals residing in other provinces of Pakistan were excluded from the study. In addition, since the survey was conducted online, those without Internet access were inherently excluded, potentially introducing digital divide bias. Beyond these criteria, the survey was open to participants of all ages, educational levels, and socioeconomic backgrounds within KPK. This broad approach allowed for a diverse and comprehensive assessment of knowledge, attitudes, and practices related to e-cigarette use across the province.

We adopted a validated questionnaire from a study done in Karachi [14] for our survey, which contained 21 items. The questionnaire had four sections, of which the first section had questions regarding demographics (Appendices). Our study utilized the age group classifications defined by the United Nations Statistics Division, as outlined in Table 1.

**Table 1:** Age group classification according to the United Nations Statistics Division

Age groups	Age range (years)
Young adults	18-24
Middle-aged adults	24-44
Older adults	45-64
Retirement age	65 and above

Questions about e-cigarette knowledge were asked in the second section of the questionnaire, while questions about participants' attitudes and e-cigarette practices were asked in the third and fourth sections, respectively (Appendices). Participants were asked a series of knowledge-related questions on e-cigarettes, including whether they knew anything about them, their contents, how much nicotine they contained, and where they learned this information. The participants' attitudes were evaluated using four- and five-point Likert scales. The questionnaire also had questions in which the comparison between normal cigarettes' effects and addiction and those of e-cigarettes was assessed. Practice-related questions assessed whether participants had ever used e-cigarettes or had used them within the past 30 days. Queries related to the patients' socioeconomic status were used to classify them into upper, upper middle, lower middle, upper lower, and lower classes, based on a modified version of the Kuppuswamy socioeconomic status scale, with scores ranging from below 5 to 29. This scale has been validated and approved for use in Pakistan (Appendices) [21].

Data were entered and analyzed using IBM SPSS Statistics version 20.0 (IBM Corp., Armonk, NY). Descriptive analyses were conducted using frequencies and tables to summarize participant responses. The chi-square test was applied to examine associations between knowledge, attitudes, and practices related to e-cigarette use and demographic variables such as gender and educational level. Statistical significance was set at a p-value < 0.05. Data collection was carried out through Google Forms, which mandated responses to all questions before submission, ensuring that the dataset was complete. After collection, the data were carefully handled and stored to maintain accuracy and protect participant privacy.

## Results

**Figure 1:**
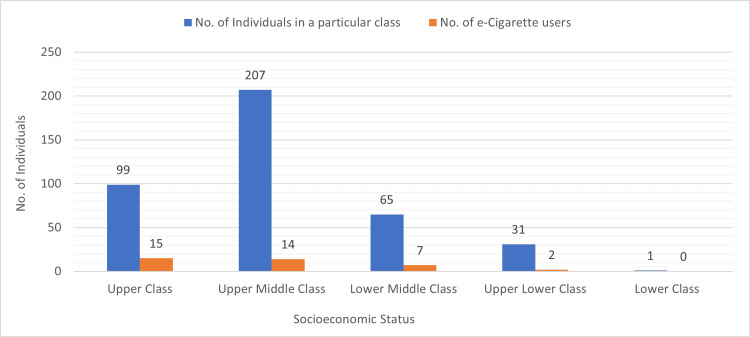
E-cigarette use and socioeconomic status

Of the 403 individuals who completed the survey, 254 (63%) were men and 149 (37%) were women. The participants' mean age was 23.6±5.4 years, with young adults making up the majority (n=290, 72%). Most of the respondents (n=296, 73.4%) were educated up to bachelor's or master's, and most of them (n=216, 53.6%) were unemployed. Most of the respondents (n=207, 51.4%) belonged to the upper middle class, whereas 99 (24.6%) belonged to the upper class. In our survey, only 38 (9.4%) individuals admitted to being e-cigarette users. Out of these, most of them belonged to the upper class (n=15) and upper middle class (n=14). These results are shown in Figure 1.

Most of the participants (n=340, 84.4%) were aware of what e-cigarettes were, and half (n=196, 48.6%) obtained that information from friends. Furthermore, we discovered that while the majority of the respondents (n=228, 56.6%) were aware of the numerous chemicals and substances included in e-cigarettes, they were unaware of the varying nicotine concentrations. Most men and women understood what e-cigarettes were and the different components they contained, but men learned about them from friends, while women learned about them online. In terms of education, a higher proportion of bachelor's and intermediate students were familiar with the definition of e-cigarettes, as well as their different components and compounds. Notably, 117 (29%) individuals reported that their primary motivation for using e-cigarettes was "quitting regular smoking," while approximately half of the participants (n=126, 31.3%) said that people started smoking for recreational purposes. The majority of participants (n=211, 52.4%) were unaware of how well e-cigarettes work to assist people in stopping smoking traditional cigarettes. Of the participants, 134 (33.3%) thought that e-cigarettes were just as addictive and damaging as traditional cigarettes, whereas almost one-third (n=179, 44.4%) disputed that people harm themselves when they smoke them. A total of 207 (51.4%) respondents said that people started smoking e-cigarettes to "fit in" or "feel cool," and around half of the respondents (n=198, 49.1%) firmly agreed that all tobacco products are dangerous. The majority of respondents (n=192, 47.6%) thought that purchasing e-cigarettes was extremely difficult for young people. The majority of respondents (n=216, 53.6%) said they "definitely will not" try e-cigarettes even if a close friend asked them to, and some (n=311, 77.2%) did not think they would even with their guardian's approval. Notably, roughly one-third of the respondents (n=139, 34.5%) had tried e-cigarettes at least once in their lives. However, a resounding majority (n=365, 90.6%) denied ever having used an e-cigarette. The majority of respondents (n=235, 58.3%) claimed not to live with or be around people who encouraged e-cigarette use. E-cigarette use is not something that most participants (n=379, 94%) would encourage or advocate. The association between the knowledge, attitude, and practices of e-cigarettes with gender and level of education is given in Tables 2, 3.

**Table 2:** Comparison between knowledge, attitudes, and practices, and gender We utilized the chi-square test to analyze associations between categorical variables in our study. A p-value of ≤0.05 was considered statistically significant.

Questions	Responses	Gender		P-values
		Male	Female	
1. Do you know what e-cigarettes are?	Yes	220 (54.6%)	120 (29.8%)	0.105
	No	34 (8.4%)	29 (7.2%)	
2. If yes, then how did you learn about them?	Internet	76 (18.8%)	77 (19.1%)	0.000
	Friends	149 (36.9%)	47 (11.7%)	
	Television	11 (2.7%)	10 (2.5%)	
	Magazines	4 (0.9%)	2 (0.5%)	
	Newspapers	2 (0.5%)	0 (0.0%)	
3. Are you aware of the various ingredients and chemicals in e-cigarette smoke?	Yes	152 (37.7%)	76 (18.9%)	0.084
	No	102 (25.3%)	73 (18.1%)	
4. Do you know about the different levels of nicotine in e-cigarettes?	Yes	78 (19.3%)	43 (10.7%)	0.696
	No	176 (43.7%)	106 (26.3%)	
5. What do you think makes people start smoking e-cigarettes?	Stress	28 (6.9%)	24 (5.9%)	0.157
	Depression	34 (8.4%)	25 (6.2%)	
	Peer pressure	23 (5.3%)	19 (4.7%)	
	Acceptability in the family	3 (0.7%)	4 (0.9%)	
	Recreational use	86 (21.3%)	40 (9.9%)	
	To quit regular smoking	80 (19.8%)	37 (9.18%)	
6. How effective do you think e-cigarettes are in helping people quit smoking regular cigarettes?	Extremely effective	9 (2.2%)	12 (2.9%)	0.022
	Slightly effective	11 (2.7%)	4 (0.9%)	
	No idea	123 (30.5%)	88 (21.8%)	
	Slightly ineffective	86 (21.3%)	32 (7.9%)	
	Extremely ineffective	25 (6.2%)	13 (3.2%)	
7. What do you think about the statement "people harm themselves when smoking e-cigarettes"?	Strongly agree	60 (14.9%)	54 (13.4%)	0.037
	Agree	115 (28.5%)	64 (15.9%)	
	Undecided	57 (14.1%)	25 (6.2%)	
	Disagree	20 (4.9%)	6 (1.5%)	
	Strongly disagree	2 (0.5%)	0 (0%)	
8. When comparing the harmful effects of e-cigarettes to regular cigarettes, how harmful do you think they are?	Much more harmful	10 (2.5%)	5 (1.2%)	0.525
	Slightly more harmful	89 (22%)	42 (10.4)	
	Equally harmful	79 (19.6%)	55 (13.6%)	
	Slightly less harmful	35 (8.7%)	18 (4.5%)	
	Much less harmful	41 (10.2%)	29 (7.2%)	
9. When comparing the addiction of e-cigarettes to regular cigarettes, how addictive do you think they are?	Much more addictive	8 (1.9%)	5 (1.2%)	0.063
	Slightly more addictive	78 (19.3%)	34 (8.4%)	
	Equally addictive	77 (19.1%)	58 (14.4%)	
	Slightly less addictive	45 (11.2%)	16 (3.9%)	
	Much less addictive	46 (11.4%)	36 (8.9%)	
10. What do you think about the statement "all tobacco products are dangerous"?	Strongly agree	112 (27.8%)	86 (21.3%)	0.076
	Agree	110 (27.3%)	50 (12.4%)	
	Undecided	21 (5.2%)	6 (1.5%)	
	Disagree	7 (1.7%)	5 (1.2%)	
	Strongly disagree	4 (0.9%)	2 (0.5%)	
11. Do you think smoking e-cigarettes makes young people "fit in," feel "cool," and become socially more acceptable?	Yes	140 (34.7%)	67 (16.6%)	0.049
	No	114 (28.3%)	82 (20.3%)	
12. How easy do you think it is to buy e-cigarettes for young people of your age?	Very easy	130 (32.3%)	62 (15.4%)	0.237
	Easy	104 (25.8%)	69 (17.1%)	
	Difficult	15 (3.7%)	13 (3.2%)	
	Very difficult	5 (1.2%)	5 (1.2%)	
13. If a good friend of yours wanted you to try e-cigarettes, would you try them?	Definitely will	13 (3.2%)	3 (0.7%)	0.000
	Probably will	51 (12.6%)	13 (3.2%)	
	Probably will not	77 (19.1%)	30 (7.4%)	
	Definitely will not	113 (28%)	103 (25.6%)	
14. If you ever try smoking e-cigarettes, do you think you would do it with your guardian's permission?	Yes	52 (12.9%)	40 (9.9%)	0.141
	No	202 (50.1%)	109 (27%)	
15. Are you surrounded by or live with people who use and promote the use of e-cigarettes?	Yes	120 (29.8%)	48 (11.9%)	0.003
	No	134 (33.2%)	101 (25%)	
16. Do you or have you ever smoked e-cigarettes (even one or two puffs)?	Yes	114 (28.3%)	25 (6.2%)	0.000
	No	140 (34.7%)	124 (30.8%)	
17. Are you a current e-cigarette smoker (smoked in the past 30 days)?	Yes	30 (7.4%)	8 (1.9%)	0.033
	No	224 (55.6%)	141 (34.9%)	
18. Would you ever promote or recommend the use of e-cigarettes to other people if you got the chance to?	Yes	16 (3.9%)	8 (1.9%)	0.703
	No	238 (59%)	141 (34.9%)	

**Table 3:** Comparison between knowledge, attitudes, and practices, and level of education We utilized the chi-square test to analyze associations between categorical variables in our study. A p-value of ≤0.05 was considered statistically significant.

Questions	Responses	Level of education				P-values
		Doctorate	Bachelors	Intermediate	Matric	
1. Do you know what e-cigarettes are?	Yes	22 (5.5%)	256 (63.5%)	58 (14.4%)	4 (0.9%)	0.299
	No	7 (1.7%)	46 (11.4%)	8 (1.9%)	2 (0.5%)	
2. If yes, then how did you learn about them?	Internet	8 (1.9%)	112 (27.8%)	30 (7.4%)	3 (0.7%)	0.002
	Friends	13 (3.2%)	151 (37.5%)	29 (7.2%)	3 (0.7%)	
	Television	4 (0.9%)	15 (3.7%)	2 (0.5%)	0 (0.0%)	
	Magazines	1 (0.2%)	4 (0.9%)	1 (0.2%)	0 (0.0%)	
	Newspapers	2 (0.5%)	0 (0.0%)	0 (0.0%)	0 (0.0%)	
3. Are you aware of the various ingredients and chemicals in e-cigarette smoke?	Yes	15 (3.7%)	173 (42.9%)	37 (9.2%)	3 (0.7%)	0.929
	No	14 (3.5%)	129 (32%)	29 (7.2%)	3 (0.7%)	
4. Do you know about the different levels of nicotine in e-cigarettes?	Yes	7 (1.7%)	89 (22%)	23 (5.7%)	2 (0.5%)	0.733
	No	22 (5.5%)	213 (52.8%)	43 (10.6%)	4 (0.9%)	
5. What do you think makes people start smoking e-cigarettes?	Stress	5 (1.2%)	39 (9.7%)	8 (1.9%)	0 (0.0%)	0.044
	Depression	6 (1.5%)	42 (10.4%)	10 (2.5%)	1 (0.2%)	
	Peer pressure	3 (0.7%)	28 (6.9%)	11 (2.7%)	0 (0.0%)	
	Acceptability in the family	0 (0.0%)	5 (1.2%)	1 (0.2%)	1 (0.2%)	
	Recreational use	9 (2.2%)	87 (21.6%)	27 (6.7%)	3 (0.7%)	
	To quit regular smoking	6 (1.5%)	101 (25%)	9 (2.2%)	1 (0.2%)	
6. How effective do you think e-cigarettes are in helping people quit smoking regular cigarettes?	Extremely effective	3 (0.7%)	12 (2.9%)	4 (0.9%)	2 (0.5%)	0.065
	Slightly effective	0 (0.0%)	12 (2.9%)	3 (0.7%)	0 (0.0%)	
	No idea	15 (3.7%)	156 (38.7%)	36 (8.9%)	4 (0.9%)	
	Slightly ineffective	10 (2.5%)	94 (23.3%)	14 (3.5%)	0 (0.0%)	
	Extremely ineffective	1 (0.2%)	28 (6.9%)	9 (2.2%)	0 (0.0%)	
7. What do you think about the statement "people harm themselves when smoking e-cigarettes"?	Strongly agree	11 (2.7%)	84 (20.8%)	19 (4.7%)	0 (0.0%)	0.000
	Agree	9 (2.2%)	138 (34.2%)	29 (7.2%)	3 (0.7%)	
	Undecided	7 (1.7%)	57 (14.1%)	16 (3.9%)	2 (0.5%)	
	Disagree	2 (0.5%)	22 (5.5%)	2 (0.5%)	0 (0.0%)	
	Strongly disagree	0 (0.0%)	1 (0.2%)	0 (0.0%)	1 (0.2%)	
8. When comparing the harmful effects of e-cigarettes to regular cigarettes, how harmful do you think they are?	Much more harmful	1 (0.2%)	8 (1.9%)	5 (1.2%)	1 (0.2%)	0.459
	Slightly more harmful	8 (1.9%)	100 (24.8%)	21 (5.2%)	2 (0.5%)	
	Equally harmful	11 (2.7%)	97 (24%)	25 (6.2%)	1 (0.2%)	
	Slightly less harmful	3 (0.7%)	42 (10.4%)	8 (1.9%)	0 (0.0%)	
	Much less harmful	6 (1.5%)	55 (13.6%)	7 (1.7%)	2 (0.5%)	
9. When comparing the addiction of e-cigarettes to regular cigarettes, how addictive do you think they are?	Much more addictive	1 (0.2%)	9 (2.2%)	2 (0.5%)	1 (0.2%)	0.91
	Slightly more addictive	8 (1.9%)	85 (21%)	18 (4.5%)	1 (0.2%)	
	Equally addictive	10 (2.5%)	102 (25.3%)	21 (5.2%)	2 (0.5%)	
	Slightly less addictive	4 (0.9%)	48 (11.9%)	9 (2.2%)	0 (0.0%)	
	Much less addictive	6 (1.5%)	58 (14.4%)	16 (3.9%)	2 (0.5%)	
10. What do you think about the statement "all tobacco products are dangerous"?	Strongly agree	18 (4.5%)	150 (37.2%)	28 (6.9%)	2 (0.5%)	0.037
	Agree	7 (1.7%)	120 (29.8%)	30 (7.4%)	3 (0.7%)	
	Undecided	1 (0.2%)	22 (5.4%)	4 (0.9%)	0 (0.0%)	
	Disagree	2 (0.5%)	6 (1.5%)	4 (0.9%)	0 (0.0%)	
	Strongly disagree	1 (0.2%)	4 (0.9%)	0 (0.0%)	1 (0.2%)	
11. Do you think smoking e-cigarettes makes young people "fit in," feel "cool," and become socially more acceptable?	Yes	13 (3.2%)	155 (38.5%)	35 (8.7%)	4 (0.9%)	0.769
	No	16 (3.9%)	147 (36.5%)	31 (7.7%)	2 (0.5%)	
12. How easy do you think it is to buy e-cigarettes for young people of your age?	Very easy	0 (0.0%)	8 (1.9%)	1 (0.2%)	1 (0.2%)	0.547
	Easy	3 (0.7%)	21 (5.2%)	4 (0.9%)	0 (0.0%)	
	Difficult	13 (3.2%)	131 (32.5%)	26 (6.4%)	3 (0.7%)	
	Very difficult	13 (3.2%)	142 (34.9%)	35 (8.7%)	2 (0.5%)	
13. If a good friend of yours wanted you to try e-cigarettes, would you try them?	Definitely will	1 (0.2%)	11 (2.7%)	4 (0.9%)	0 (0.0%)	0.41
	Probably will	2 (0.5%)	49 (12.2%)	12 (2.9%)	1 (0.2%)	
	Probably will not	9 (2.2%)	75 (18.6%)	20 (4.9%)	3 (0.7%)	
	Definitely will not	17 (4.2%)	167 (41.4%)	30 (7.4%)	2 (0.5%)	
14. If you ever try smoking e-cigarettes, do you think you would do it with your guardian's permission?	Yes	7 (1.7%)	65 (16.1%)	18 (4.5%)	2 (0.5%)	0.697
	No	22 (5.5%)	237 (58.8%)	48 (11.9%)	4 (0.9%)	
15. Are you surrounded by or live with people who use and promote the use of e-cigarettes?	Yes	11 (2.7%)	126 (31.2%)	28 (6.9%)	3 (0.7%)	0.95
	No	18 (4.5%)	176 (43.7%)	38 (9.4%)	3 (0.7%)	
16. Do you or have you ever smoked e-cigarettes (even one or two puffs)?	Yes	6 (1.5%)	105 (26%)	25 (6.2%)	3 (0.7%)	0.33
	No	23 (5.7%)	197 (48.9%)	41 (10.2%)	3 (0.7%)	
17. Are you a current e-cigarette smoker (smoked in the past 30 days)?	Yes	2 (0.5%)	30 (7.4%)	5 (1.2%)	1 (0.2%)	0.815
	No	27 (6.7%)	272 (67.5%)	61 (15.1%)	5 (1.2%)	
18. Would you ever promote or recommend the use of e-cigarettes to other people if you got the chance to?	Yes	3 (0.7%)	16 (3.9%)	3 (0.7%)	2 (0.5%)	0.023
	No	26 (6.5%)	286 (70.9%)	63 (15.6%)	4 (0.9%)	

## Discussion

One of the key findings of our study was that while the majority of the participants demonstrated a basic awareness of e-cigarettes and their components, their understanding was incomplete, as reflected in their generally negative attitudes toward e-cigarette use. Most people were unaware that e-cigarettes may be used to help people stop smoking. Although the proportion of people who had just tried e-cigarettes was quite large, the percentage of individuals who were current e-cigarette smokers in our study was quite small, which is very similar to the studies conducted in Karachi [14,22]. In comparison, the use of e-cigarettes and vaping is increasing in other countries. Research conducted in the USA, UK, and Canada has revealed a rise in e-cigarette use over the last 10 years, particularly among young people. A study conducted in Eastern and Central Europe discovered that the use of e-cigarettes and vaping among teenagers had increased by 24.4% [23]. This deviation from the global average could be attributed to a number of factors, including the stigma attached to using cigarettes and other illegal drugs in Pakistani society, the majority of people's strong religious convictions that forbid using cigarettes and other drugs, or the fact that e-cigarette use is underreported due to a lack of research.

We also found that both men and women had similar views regarding e-cigarette use. Both male and female respondents held similarly negative views of e-cigarettes, largely due to limited awareness of their composition and intended use in smoking cessation. The majority of them believed that vaping and e-cigarettes were either as bad or perhaps more harmful than regular cigarettes. Both genders had limited knowledge regarding e-cigarettes since the majority of them did not know the levels of nicotine in these products, and most of them were unaware of their use in helping people quit smoking, which is one of the main motivations behind using e-cigarettes [24]. This lack of knowledge might be the reason for the negative attitude toward e-cigarettes and also the reason for their limited use in Khyber Pakhtunkhwa. Men made up the bulk of the present e-cigarette users in our survey, which is consistent with a US study that discovered that men are more likely than women to use e-cigarettes [25]. This difference may be because, in Pakistani culture, smoking and drug use are considered taboo for women and they are judged more harshly compared to men.

In our study, the vast majority of current e-cigarette users were young adults from upper-class and upper-middle-class backgrounds. A lot of them were bachelor's and intermediate-level students. This result is consistent with another study that discovered that those with higher socioeconomic levels were more likely to use e-cigarettes and were exposed to more e-cigarette commercials [26]. The higher socioeconomic level was linked to increased exposure to e-cigarette advertisements and increased e-cigarette consumption, according to a similar finding from another US study [27]. Another study also revealed that e-cigarettes are more likely to be sold in wealthy neighborhoods than in underprivileged ones [26]. Furthermore, 99% of youngsters were exposed to one or more e-cigarette advertisements, according to the same study that brought attention to the concerning levels of exposure of young adults to these advertisements. Therefore, the increased exposure of the youth and upper socioeconomic classes to e-cigarette-related information and advertisements may be one of the primary causes of the popularity of e-cigarettes and vaping in Pakistani culture.

Our study was limited to the province of Khyber Pakhtunkhwa; therefore, further studies should be carried out in other provinces, cities, and lower-income areas of Pakistan to help bridge the knowledge gap nationwide. Additionally, our research was subject to digital divide bias, as individuals without Internet access may have been excluded due to the online nature of the survey. The use of non-probability convenience sampling also introduces selection bias, which may limit the generalizability of our findings to the broader population. It would be beneficial to investigate the efficacy of e-cigarettes in aiding individuals to give up traditional smoking and whether people who started smoking e-cigarettes to quit regular smoking succeeded in doing so or not. Additional research is needed to determine the prevalence of e-cigarette use and people's attitudes about it across a range of age groups, genders, and socioeconomic statuses. Extensive research should be conducted on the potential risks, benefits, and long-term impacts of e-cigarettes on human health in order to appropriately regulate these devices. Furthermore, research ought to be done on how advertising and e-cigarette promotion contribute to the youth population's e-cigarette addiction, particularly in developing countries such as Pakistan.

A constraint of our research was that we did not take into account the potential negative effects of electronic cigarettes, including their harmful impact on the lungs and cardiovascular system, as well as their potential role as a gateway to conventional cigarette smoking or other substance use, particularly among youth. Our study did not assess participants' intentions regarding e-cigarette use for smoking cessation. Furthermore, long-term quit rates and motivations for e-cigarette initiation were not explored. Additionally, long-term former conventional cigarette smokers who had quit were not included in our study and therefore could not be compared to current smokers.

## Conclusions

The purpose of this study was to assess the general population's knowledge and attitudes toward e-cigarette use. Our findings indicate that most individuals lack adequate knowledge about electronic cigarettes, which is reflected in their largely negative perceptions. While male and female respondents held similar views, the majority of current users were young adults from higher socioeconomic backgrounds, possibly due to greater exposure to marketing and the social unacceptability of smoking among women in Pakistan. This study sheds light on the knowledge, attitudes, and practices (KAP) regarding e-cigarette use, an area that has not been previously explored in Khyber Pakhtunkhwa. It adds to the limited literature on e-cigarette use in Pakistan by highlighting the need for public education and targeted policy interventions. Future research should assess the role of e-cigarettes in smoking cessation, explore usage patterns across demographic groups, and evaluate the influence of marketing and long-term health impacts to guide effective regulation.

## Appendices

Questionnaire

Thank you very much for agreeing to participate in our research study. We appreciate your willingness to participate in our research. Your responses will remain anonymous and confidential, and your personal information will not be shared with any third parties. The questionnaire should take approximately five minutes to complete. Please answer the questions honestly and to the best of your knowledge, as your feedback will play a crucial role in shaping our research outcomes.

The demographic data of the study participants are shown in Table 4.

**Table 4:** Demographic data of participants

Age:_________
Gender:_________
Level of education
a. Doctorate or honors
b. Bachelor's or master's degree
c. Intermediate (FSc/FA/A levels/IB/ AP)
d. Matric (O levels)
e. Middle school
f. Primary school
g. None
Occupation
a. Professional
b. Semi-professional
c. Clerical/shop owner/farmer
d. Skilled worker
e. Semi-skilled worker
f. Unskilled worker
g. Unemployed

Table 5 presents the Kuppuswamy socioeconomic status scale.

**Table 5:** Kuppuswamy socioeconomic status scale This table presents the Kuppuswamy socioeconomic status scale. It is an open-access tool and has been reformed into a table form for clarity [28,29].

Education of the head of the family
a. Professional degree
b. Graduate or postgraduate
c. Intermediate or post-high school diploma
d. High school certificate
e. Middle school certificate
f. Primary school certificate
g. Illiterate
Occupation of the head of the family
a. Professional
b. Semi-professional
c. Clerical, shop owner, farmer
d. Skilled worker
e. Semi-skilled worker
f. Unskilled worker
g. Unemployed
Monthly income of family (in Pakistani Rupees)
a. >397,236
b. 198,468-397,235
c. 149,079-198,467
d. 99,384-149,078
e. 59,694-99,383
f. 20,001-59,693
g. <20,000

The questionnaire used in this study is shown in Table 6.

**Table 6:** Knowledge, attitude, and practices of e-cigarettes This table presents the questionnaire comprising questions on the knowledge, attitudes, and practices of e-cigarettes. For clarity, the questionnaire has been structured in a tabular format. The questionnaire items in this table were adopted from an open-access article [14].

Do you know what e-cigarettes are?
a. Yes
b. No
If yes, then how did you learn about them?
a. Internet
b. Friends
c. Television
d. Magazines
e. Newspapers
Are you aware of the various ingredients and chemicals in e-cigarette smoke?
a. Yes
b. No
Do you know about the different levels of nicotine in e-cigarettes?
a. Yes
b. No
What do you think makes people start smoking e-cigarettes?
a. Stress
b. Depression
c. Peer pressure
d. Acceptability in the family
e. Recreational use
f. To quit smoking regular cigarettes
How effective do you think e-cigarettes are in helping people quit smoking regular cigarettes?
a. Extremely effective
b. Slightly effective
c. No idea
d. Slightly ineffective
e. Extremely ineffective
What do you think about the statement "people harm themselves when smoking e-cigarettes"?
a. Strongly agree
b. Agree
c. Undecided
d. Disagree
e. Strongly disagree
When comparing the harmful effects of e-cigarettes to regular cigarettes, how harmful do you think they are?
a. Much more harmful
b. Slightly more harmful
c. Equally harmful
d. Slightly less harmful
e. Much less harmful
When comparing the addiction of e-cigarettes to regular cigarettes, how addictive do you think they are?
a. Much more addictive
b. Slightly more addictive
c. Equally addictive
d. Slightly less addictive
e. Much less addictive
What do you think about the statement "all tobacco products are dangerous"?
a. Strongly agree
b. Agree
c. Undecided
d. Disagree
e. Strongly disagree
Do you think smoking e-cigarettes makes young people "fit in," feel "cool," and become socially more acceptable?
a. Yes
b. No
How easy do you think it is to buy e-cigarettes for young people of your age?
a. Very easy
b. Easy
c. Difficult
d. Very difficult
If a good friend of yours wanted you to try e-cigarettes, would you try them?
a. Definitely will
b. Probably will
c. Probably will not
d. Definitely will not
If you ever try smoking e-cigarettes, do you think you would do it with your guardian's permission?
a. Yes
b. No
Are you surrounded by or live with people who use and promote the use of e-cigarettes?
a. Yes
b. No
Do you or have you ever smoked e-cigarettes (even one or two puffs)?
a. Yes
b. No
Are you a current e-cigarette smoker (smoked in the past 30 days)?
a. Yes
b. No
Would you ever promote or recommend the use of e-cigarettes to other people if you got the chance to?
a. Yes
b. No
